# IEEE 802.15.4 ZigBee-Based Time-of-Arrival Estimation for Wireless Sensor Networks

**DOI:** 10.3390/s16020203

**Published:** 2016-02-05

**Authors:** Jeonghyeon Cheon, Hyunsu Hwang, Dongsun Kim, Yunho Jung

**Affiliations:** 1 School of Electronics and Information Engineering, Korea Aerospace University, 76 Hanggongdaehak-ro, Goyang-si, Gyeonggi-do 412-791, Korea; jhcheon@kau.kr (J.C.); hshwang@kau.kr (H.H.); 2 Korea Electronics Technology Institute, 25 Saenari-ro, Seongnam-si, Gyeonggi-do 463-816, Korea; dskim@keti.re.kr

**Keywords:** positioning system, time-of-arrival, wireless sensor networks, ZigBee

## Abstract

Precise time-of-arrival (TOA) estimation is one of the most important techniques in RF-based positioning systems that use wireless sensor networks (WSNs). Because the accuracy of TOA estimation is proportional to the RF signal bandwidth, using broad bandwidth is the most fundamental approach for achieving higher accuracy. Hence, ultra-wide-band (UWB) systems with a bandwidth of 500 MHz are commonly used. However, wireless systems with broad bandwidth suffer from the disadvantages of high complexity and high power consumption. Therefore, it is difficult to employ such systems in various WSN applications. In this paper, we present a precise time-of-arrival (TOA) estimation algorithm using an IEEE 802.15.4 ZigBee system with a narrow bandwidth of 2 MHz. In order to overcome the lack of bandwidth, the proposed algorithm estimates the fractional TOA within the sampling interval. Simulation results show that the proposed TOA estimation algorithm provides an accuracy of 0.5 m at a signal-to-noise ratio (SNR) of 8 dB and achieves an SNR gain of 5 dB as compared with the existing algorithm. In addition, experimental results indicate that the proposed algorithm provides accurate TOA estimation in a real indoor environment.

## 1. Introduction

With the advancement of wireless sensor network (WSN) technology, application services that use location information from various sensors have been attracting considerable attention [1,2,3]. The environments for obtaining such location information can be largely divided into outdoor and indoor environments. Although outdoor positioning is possible with global positioning system (GPS) technology, new positioning technology is required for indoor positioning. As a result, various radio frequency (RF)-based positioning techniques that employ wireless network technology are being developed. In particular, because 3D positioning is possible using a triangulation scheme when the distances to four reference points are known, time of arrival (TOA)-based positioning technology is commonly used to measure distances based on the propagation time between the transmitter (TX) and the receiver (RX). Currently, ZigBee, Wi-Fi, and ultra-wide-band (UWB) systems are used for TOA estimation [4,5].

The accuracy of distance measurement using wireless network technology is a function of the signal bandwidth; using broad bandwidth is the most fundamental approach for achieving higher accuracy [6]. Hence, among the above-mentioned communication systems, UWB-based positioning technology with a bandwidth of 500 MHz has seen significant development [7,8,9,10,11,12]. According to a recent study, UWB-based positioning technology achieves highly accurate performance down to a few centimeters. However, UWB systems suffer from the disadvantages of high complexity and high power consumption due to the use of broad bandwidth. Therefore, it is difficult to employ UWB-based positioning systems in WSN applications that require low-power and low-cost implementation.

Consequently, positioning techniques that use narrow bandwidth have emerged as a popular alternative [13,14,15,16,17,18]. In particular, ZigBee, with a bandwidth of 2 MHz, is a low-power and low-cost communication system that is widely employed in WSN applications. Moreover, the ZigBee-based positioning technique offers a significant advantage in that it uses the already established infrastructure. Hence, several studies have focused on ZigBee-based positioning technology. In [15], a multi-channel distance measurement algorithm based on the frequency hopping scheme was proposed; it requires the full industrial–scientific–medical (ISM) band of 80 MHz. Even though it offers accuracy down to a meter, it requires a special phase tracking unit for preserving the phase relationship when hopping from one channel to another. Furthermore, the channel capacity is limited, owing to the use of the full ISM band. In [16], a Wiener filter was used for 16× over-sampled sequences in order to overcome the lack of bandwidth in ZigBee. Although this approach offers accuracy down to a meter, the power consumption at the sampling rate of 32 MHz and the operational complexity of the Wiener filter are considerably high. Further, the code modulus synchronization (CMS) algorithm was proposed in [17] to provide accuracy down to a meter. However, this algorithm uses a new sequence, and therefore, it is not compatible with the IEEE 802.15.4 standard [19]. In [18], a TOA estimation algorithm was proposed on the basis of auto- and cross-correlation values for the received signal and preamble. This approach is a good candidate for ZigBee-based positioning systems because it is compatible with the IEEE 802.15.4 standard and does not require additional bandwidth or complex operations.

This paper proposes a precise TOA estimation algorithm for ZigBee systems, which can estimate the TOA within the sampling interval, and presents some experimental results. The remainder of this paper is organized as follows. Section 2 explains the packet structure for TOA estimation. Section 3 describes the proposed TOA estimation algorithm. Section 4 and Section 5 present the simulation results and experimental results, respectively. Finally, Section 6 concludes the paper.

## 2. Packet Structure for TOA Estimation

Methods for measuring the distance between two devices (nodes) are commonly classified into one-way ranging (OWR) and two-way ranging (TWR) [20,21,22]. The OWR scheme is based on one transmission of a signal between TX and RX, and timing synchronization between TX and RX is assumed. Unlike OWR, the TWR scheme is based on two transmissions of a signal between TX and RX, and timing synchronization is not required. Regardless of timing synchronization, the proposed algorithm can accurately estimate the TOA. Moreover, both OWR and TWR schemes require exact TOA estimation. Therefore, the proposed TOA estimation algorithm can be applied to both schemes, and we consider the OWR scheme for convenience of explanation.

**Figure 1:**
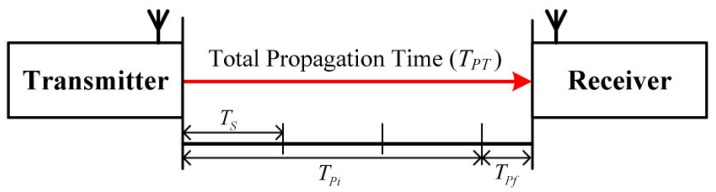
Configuration of total propagation time $T_{PT}$ of RF signal.

In the OWR scheme, the TOA estimator measures the propagation time $T_{PT}$, which is equal to TOA of the RF signal from TX to RX, as shown in Figure 1.

At RX, the cross-correlation between the received signal and the preamble is calculated at every sampling period $T_{s}$ and the reception timing is determined as that when the cross-correlation value is maximal. In the case of IEEE 802.15.4 ZigBee systems that use a sampling rate of 8 MHz with 4× over-sampling, the measured distance error reaches ±18.25 m. Therefore, the TOA within the sampling interval needs to be measured to increase the estimation accuracy. Accordingly, the total propagation time $T_{PT}$ consists of the integer-part time $T_{Pi}$, which is estimated at the sampling interval, and the fraction-part time $T_{Pf}$, which is estimated within the sampling interval:

(1) $$T_{PT}=T_{Pi}+T_{Pf}$$

**Figure 2:**
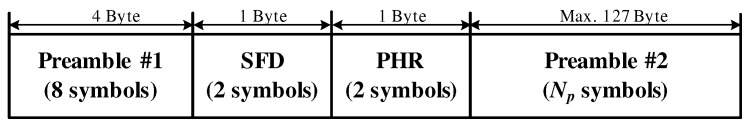
Packet structure for time of arrival (TOA) estimation. SFD: start-of-frame delimiter; PHR: PHY header.

The packet structure for TOA estimation is based on the IEEE 802.15.4 ZigBee standard as shown in Figure 2. Here, preamble #1 consists of 8× repeated index-0 symbols ($S_{0}$’s), which includes 32 chips according to the IEEE 802.15.4 standard, and $T_{Pi}$ is calculated using this preamble. Further, preamble #2 is defined for the estimation of $T_{Pf}$, which consists of $N_{P}\times$ repeated $S_{0}$’s. As defined in the IEEE 802.15.4 standard, the start-of-frame delimiter (SFD), which consists of two symbols, $S_{7}$ and $S_{10}$, is a field that indicates the end-timing of preamble #1. In addition, PHY header (PHR) contains frame-length information.

## 3. Proposed ToA Estimation Algorithm

### 3.1. $T_{Pi}$ estimation

**Figure 3:**
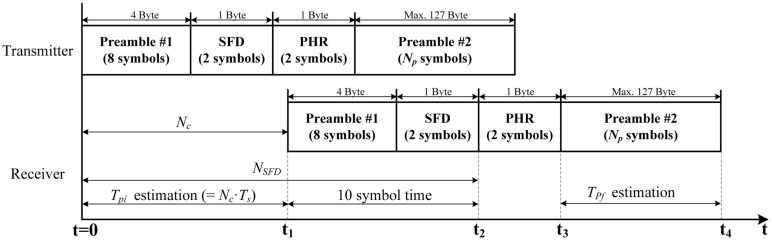
TOA integer-part estimation.

TOA integer-part time $T_{Pi}$ can be estimated by the product of the count value $N_{c}$ and the sampling period $T_{s}$:

(2) $$T_{Pi}=N_{c}\cdot T_{s}$$

where $N_{c}$ is the number of samples from the transmit timing to the receive timing. Actually, because preamble #1 consists of the 8× repeated symbols and the time synchronization for the received packet is completed at a random point in time, the receiver cannot know the exact start timing of preamble #1. Therefore, $N_{c}$ is calculated by subtracting the count value that corresponds to ten-symbol time from $N_{SFD}$, which is the number of samples until the instant that SFD is detected, as shown in Figure 3. Therefore, exact time synchronization and SFD detection is crucial for estimating $T_{Pi}$.

On the other hand, there exists a maximum carrier frequency offset (CFO) of ±40 ppm between TX and RX, as defined in the IEEE 802.15.4 standard. Since time synchronization and SFD detection are based on the cross-correlation value and the cross-correlation is distorted by CFO, an algorithm that is robust against CFO is required. In particular, because the degradation by CFO is proportional to the number of samples, robust correlation is crucial for long packets such as the TOA packet. Moreover, because low-complexity implementation is required for WSN applications, non-coherent detection should be applied instead of coherent detection. Therefore, the proposed TOA estimator uses the double-correlation algorithm [23,24], which is known to be very robust against CFO. At the receiver, the received signal r(n) is given by

(3) $$r\left(n\right)=s_{k}\left(n\right)e^{j\omega_{0}n+\theta }+w\left(n\right)$$

where $s_{k}\left(n\right)$ is the transmitted signal whose symbol index is *k* (k∈{0,1,...,15}), $\omega_{0}$ is the CFO, *θ* is the initial phase offset, and w(n) is the additive white Gaussian noise (AWGN). The double-correlation value for the *n*-th sample can be expressed as

(4) $$r\left(n\right)=s_{k}\left(n\right)e^{j\omega_{0}n+\theta }+w\left(n\right)$$

(5) $$\begin{array}{c} C_{DC}\left(n\right)=\sum_{m=0}^{N_{s}-1}r^{*}\left(n+m\right)r\left(n+m-N_{D}\right)\cdot s_{0}\left(n+m\right)s_{0}^{*}\left(n+m-N_{D}\right)\\ =\sum_{m=0}^{N_{s}-1}s_{k}^{*}\left(n+m\right)e^{-j\omega_{0}\left(n+m\right)-\theta }s_{k}\left(n+m-N_{D}\right)e^{j\omega_{0}\left(n+m-N_{D}\right)+\theta }\cdot s_{0}\left(n+m\right)s_{0}^{*}\left(n+m-N_{D}\right)+w^{\prime }\left(n\right)\\ =e^{-j\omega_{0}N_{D}}\sum_{m=0}^{N_{s}-1}s_{k}^{*}\left(n+m\right)s_{k}\left(n+m-N_{D}\right)\cdot s_{0}\left(n+m\right)s_{0}^{*}\left(n+m-N_{D}\right)+w^{\prime }\left(n\right) \end{array}$$

where *m* is the sample index in the correlation window, $N_{s}$ is the total number of samples of one symbol, and $N_{D}$ is the number of delayed samples to compensate for the effect of CFO, which is set to 4 in the case of 4× over-sampling. As shown in Equation (4), the initial phase offset is eliminated, which means that non-coherent detection is possible. In addition, the effect of CFO decreases to a constant value, which indicates that it is not proportional to the number of samples.

### 3.2. $T_{Pf}$ Estimation

**Figure 4:**
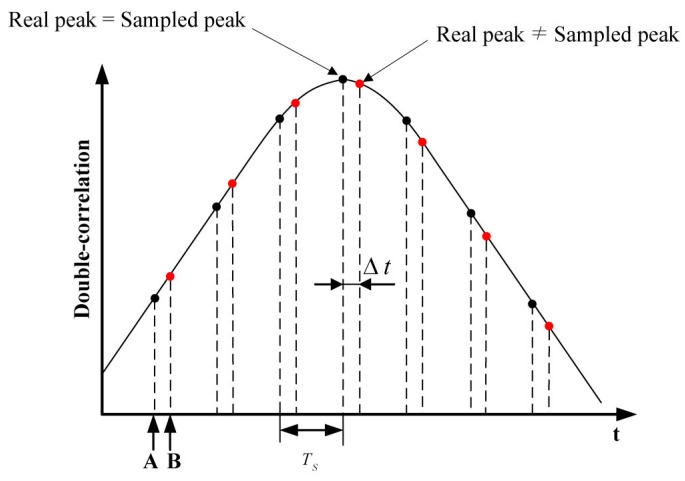
Timing offset between real peak and sampled peak for correlation values.

As shown in Figure 4, there exists a timing offset (Δt) between the peak values of double-correlation when the sampling process starts.

In the case of **A** in Figure 4, the real peak is the same as the sampled peak. However, there exists an offset in the case of **B**. This timing offset causes the measured distance error, which reaches ±18.25 m as mentioned earlier; therefore, it should be estimated for precise TOA estimation. Moreover, this offset is equal to $T_{Pf}$ because $T_{Pi}$ is calculated on the basis of the double-correlation value.

In order to estimate $T_{Pf}$, the proposed TOA estimator uses preamble #2, which consists of $N_{P}\times$ repeated $S_{0}$’s. First, the double-correlation value is accumulated to eliminate the effect of AWGN, and the accumulated $C_{DC}$ is given by

(6) $$C\left(u\right)=\sum_{l=0}^{N_{p}-1}C_{DC}\left((N_{s}-1)l+u\right)$$

where *u* denotes the sample index for the accumulated double-correlation value. When *u* is equal to *p*, which is the peak timing of the double-correlation value, C(u=p) is given by

(7) $$C\left(p\right)=\sum_{l=0}^{N_{p}-1}C_{DC}\left((N_{s}-1)l+p\right)$$

**Figure 5:**
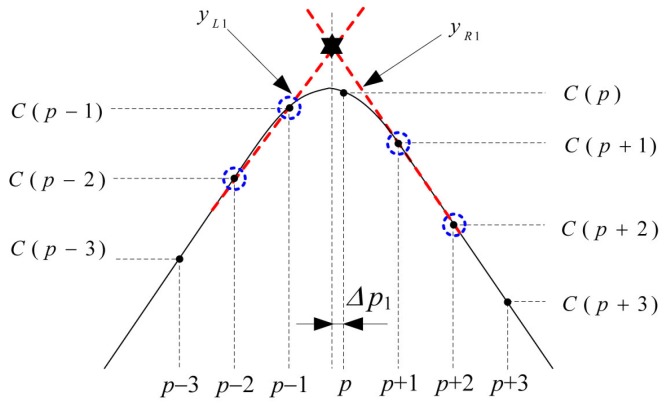
Estimation of $\Delta p_{1}$ using C(p±1) and C(p±2).

**Figure 6:**
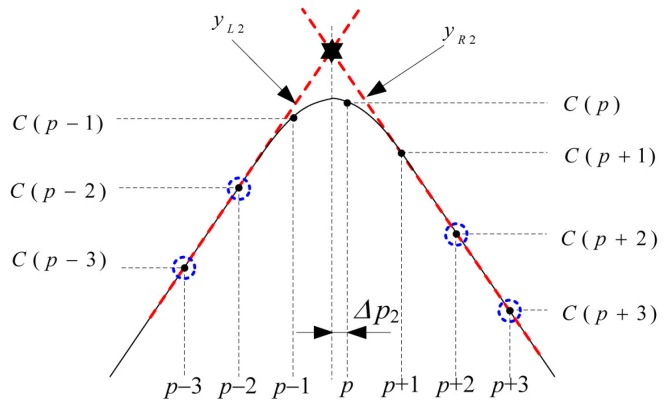
Estimation of $\Delta p_{2}$ using C(p±2) and C(p±3).

The proposed algorithm estimates $T_{Pf}$ from the intersection of the linear equations on the left-hand side and right-hand side of the double-correlation value, as shown in Figure 5 and Figure 6. First, two line equations, $y_{L1}$ and $y_{R1}$, are obtained from C(p±1) and C(p±2). Using their intersection point, the first timing offset $\Delta p_{1}$ within the sample interval is estimated as

(8) $$\Delta p_{1}=\frac{-2C(p-1)+C(p-2)+2C(p+1)-C(p+2)}{C(p-1)-C(p-2)+C(p+1)-C(p+2)}$$

Similarly, using two equations, $y_{L2}$ and $y_{R2}$, obtained from C(p±2) and C(p±3), the second timing offset $\Delta p_{2}$ is calculated as

(9) $$\Delta p_{2}=\frac{-3C(p-2)+2C(p-3)+3C(p+2)-2C(p+3)}{C(p-2)-C(p-3)+C(p+2)-C(p+3)}$$

In summary, the timing offset within the sample interval, $\Delta p_{k}$, which is estimated using the intersection point of the linear equations obtained from the two consecutive points on the left-hand side and right-hand side of C(p), can be expressed as

(10) $$\Delta p_{k}=\frac{-(k+1)C(p-k)+(k)C(p-k-1)+(k+1)C(p+k)-(k)C(p+k+1)}{C(p-k)-C(p-k-1)+C(p+k)-C(p+k+1)}$$

Using $\Delta p_{k}$, the average timing offset Δp can be calculated as

(11) $$\Delta p=\frac{1}{L}\sum_{k=1}^{L}\Delta p_{k}$$

where *L* denotes the number of calculated timing offsets within the sample interval. Finally, $T_{Pf}$ is given by

(12) $$T_{Pf}=\Delta p\cdot T_{s}$$

## 4. Simulation Results

**Figure 7:**
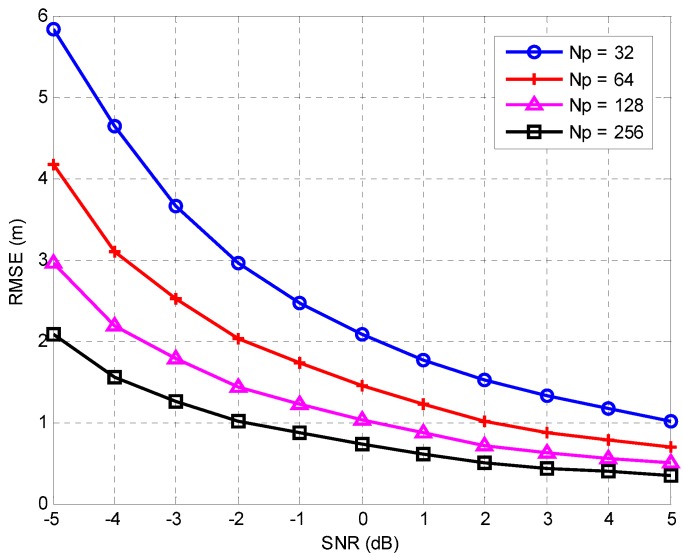
Performance of proposed algorithm for $N_{p}$ = 32, 64, 128, and 256. RMSE: root-mean-square error; SNR: signal-to-noise ratio.

The performance of the proposed TOA estimation algorithm was evaluated by computing the root-mean-square error (RMSE) for the estimated results in an environment with a CFO of ±40 ppm as well as AWGN. Figure 7 shows the performance evaluation results of the proposed TOA estimation algorithm for $N_{p}=32$, 64, 128, and 256. As $N_{p}$ increases, the degradation due to white Gaussian noise is reduced, and therefore, the RMSE values decrease.

**Figure 8:**
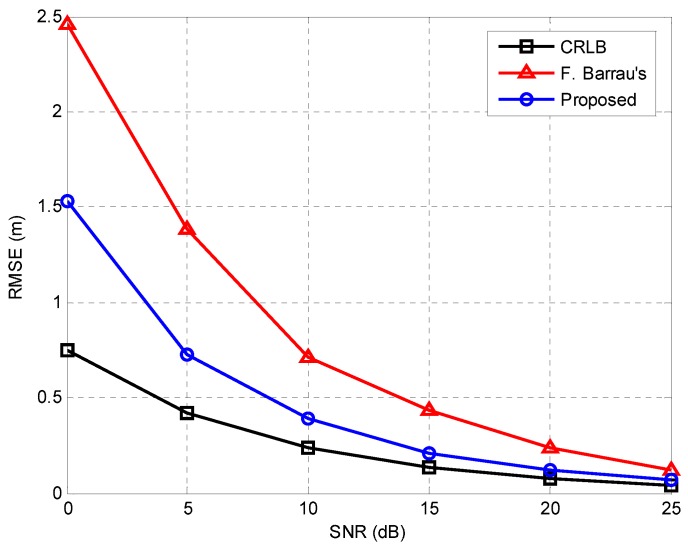
Performance comparison of proposed algorithm and F. Barrau’s algorithm [18]. CRLB: Cramer-Rao lower bound, presented for comparison.

Figure 8 shows the performance comparison results of the proposed algorithm and F. Barrau’s algorithm [18] when $N_{p}=64$, which is applicable to ZigBee systems because it is compatible with the IEEE 802.15.4 standard and does not require additional bandwidth or complex operations such as the Wiener filter. The Cramer-Rao lower bound (CRLB) [6] is also presented for comparison. As shown in this figure, the proposed algorithm provides an accuracy of 0.5 m at an SNR of 8 dB and achieves an SNR gain of around 5 dB for RMSE = 0.5 m as compared with F. Barrau’s algorithm [18]. Moreover, the performance of the proposed algorithm is close to CRLB for SNR ≥20 dB.

## 5. Experimental Results

**Figure 9:**
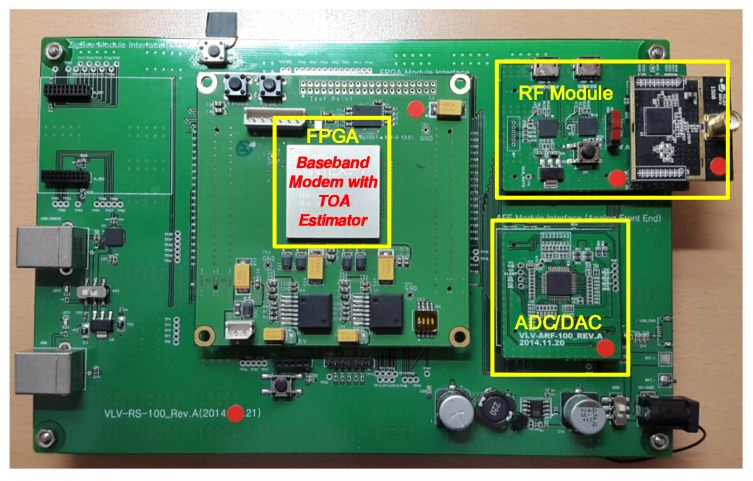
Experimental platform. FPGA: field-programmable gate array; ADC/DAC: analog-to-digital converter/digital-to-analog converter.

**Figure 10:**
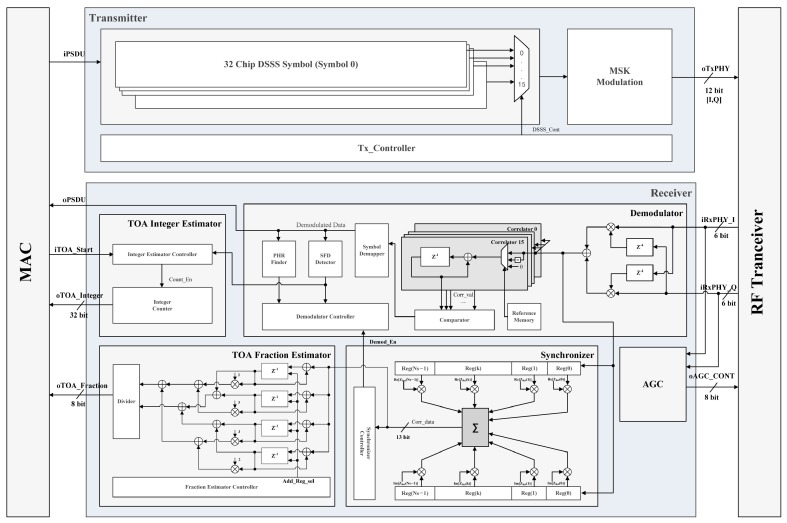
Block diagram of ZigBee baseband modem with proposed TOA estimator.

Experiments for distance measurement were conducted to verify the performance of the proposed TOA estimation algorithm. The experimental platform consists of an RF module, an analog-to-digital converter/digital-to-analog converter (ADC/DAC), and a baseband modem with a TOA estimator, as shown in Figure 9. The RF module up/down-converts the baseband signal to/from the frequency band of 2.4 GHz, which is compatible with the IEEE 802.15.4 standard. The sampling rate of the ADC/DAC is 8 MHz, which is four times the baseband signal bandwidth of 2 MHz. As shown in Figure 10, the baseband modem consists of a transmitter and receiver, and it is implemented using field-programmable gate array (FPGA). The transmitter modulates 32-chip direct-sequence spread-spectrum (DSSS) symbols with the minimum shift keying (MSK) scheme, which is also compatible with the IEEE 802.15.4 standard. The receiver consists of an automatic gain controller (AGC), a synchronizer, a demodulator, and a TOA estimator. The AGC controls the gain of the amplifiers in the RF transceiver to provide a constant amplitude for the received signal. The synchronizer finds the DSSS symbol boundary using the double-correlation value. Further, the demodulator detects the transmitted symbol, which is also based on the double-correlation value. Finally, the TOA estimator calculates the integer-part and fraction-part of the TOA using the proposed algorithm.

**Figure 11:**
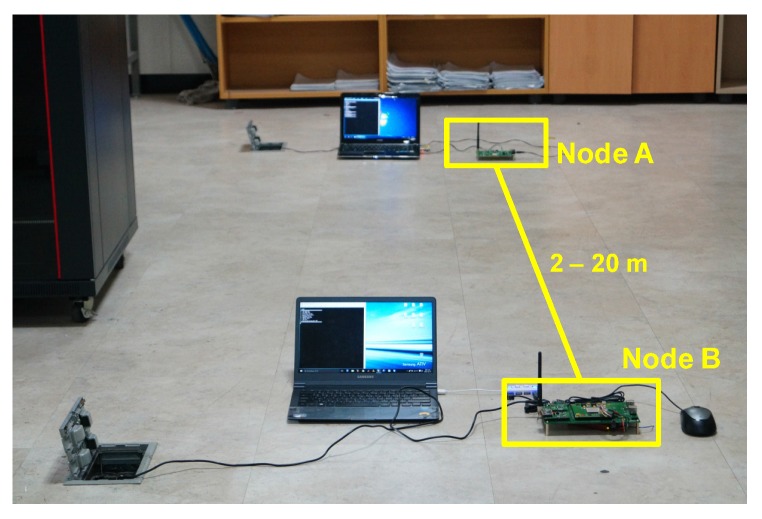
Evaluation environment in indoor experiments.

**Figure 12:**
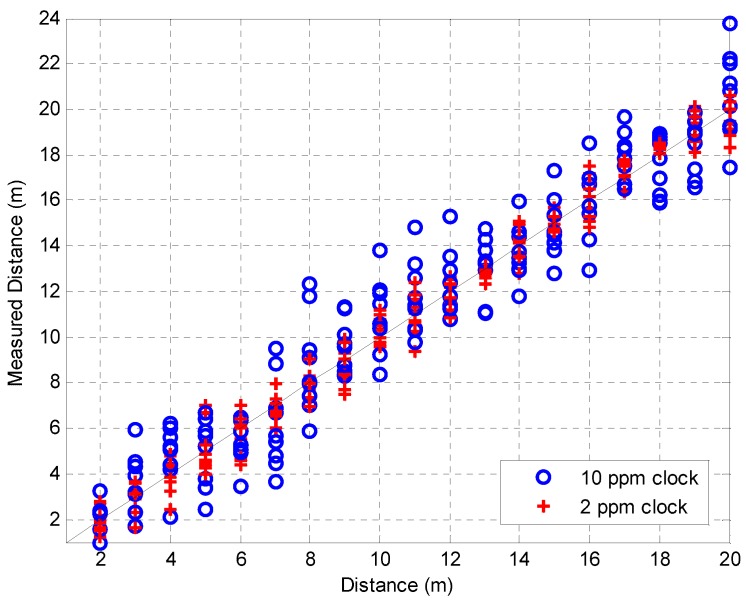
Measured distance for clock deviation.

**Figure 13:**
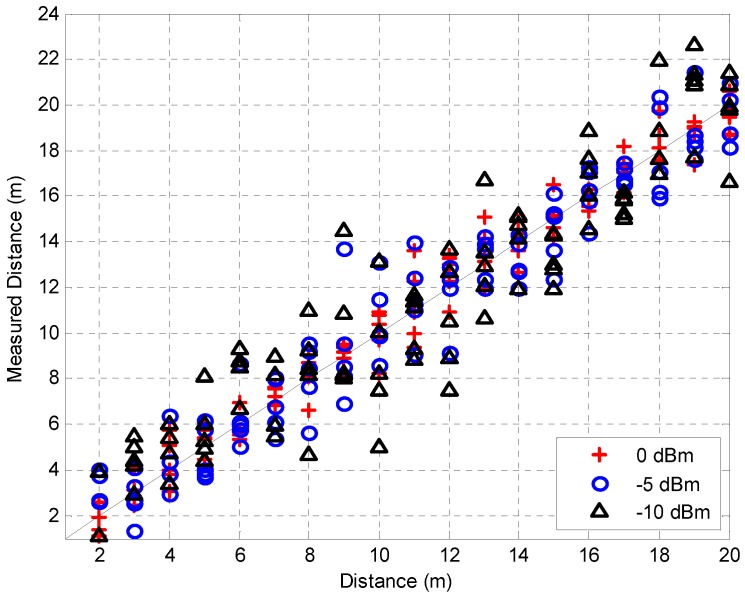
Measured distance for various transmit powers.

The experiments were performed in a WiFi-free indoor location, as shown in Figure 11, and the TWR scheme was considered because of the timing synchronization issue. Figure 12 shows the distance measurement results, which were obtained for distances of 2 m to 20 m at intervals of 1 m, and each measurement was performed ten times. The transmitter power was 10 dBm and two types of crystal oscillators (clock generator) were used, whose deviations were 2 ppm and 10 ppm, respectively. The RMSE values for the experimental results were 0.68 m and 1.74 m, respectively. As expected, the performance in the case of the 2 ppm clock was superior to that in the case of the 10 ppm clock. Furthermore, we can see that the performance of the 2 ppm clock was slightly degraded owing to the clock deviation as compared with the simulation results, considering that the SNR at 20 m was around 25 dB. Therefore, it was confirmed that a compensation scheme for clock deviation needs to be adopted in order to increase the ranging performance, such as symmetrical double-sided two-way ranging (SDS-TWR) [25,26]. Figure 13 shows the distance measurement results for various SNRs with a 2 ppm clock generator. In order to change the SNR, the transmitter power was set to 0 dBm, −5 dBm, and −10 dBm, and the RMSE values were 1.02 m, 1.61 m, and 2.03 m, respectively. It was also confirmed that the RMSE values increase as the SNR decreases.

## 6. Conclusions

This paper proposed a precise TOA estimation algorithm using an IEEE 802.15.4 ZigBee system with a narrow bandwidth of 2 MHz. In order to overcome the lack of bandwidth in such a system, the proposed algorithm compensates for the TOA integer-part, which is estimated at the sampling interval, with the TOA fraction-part, which is estimated within the sampling interval. The simulation results showed that the proposed algorithm can provide an accuracy of 0.5 m at an SNR of 8 dB when $N_{p}=64$, and the experimental results indicated that the proposed algorithm can provide accurate TOA estimation in a real environment. Thus, the proposed algorithm is an effective solution for positioning systems in WSN applications.
